# Broadcasters, receivers, functional groups of metabolites and the link to heart failure progression using polygenic factors

**DOI:** 10.21203/rs.3.rs-3246406/v1

**Published:** 2023-08-18

**Authors:** Azam Yazdani, Raul Mendez-Giraldez, Akram Yazdani, Daniel Schaid, Sek Won Kong, Mohamad Hadi, Ahmad Samiei, Clemens Wittenbecher, Jessica Lasky-Su, Clary Clish, Francesco Marotta, Michael Kosorok, Samia Mora, Jochen Muehlschlegel, Daniel Chasman, Martin Larson, Sarah Elsea

**Affiliations:** Brigham & Women’s Hospital; National Institute of Environmental Health Sciences, Durham; Division of Clinical and Translational Sciences, Department of Internal Medicine, at The University of Texas Health Science Center at Houston, McGovern Medical School; Department of Quantitative Health Sciences, Mayo Clinic, Rochester, MN 55902,; Boston Children’s Hospital; School of Mathematics, University of science and technology of Iran, Tehran; Computational Health Informatics Program, Boston Children’s Hospital, Boston, MA; Harvar T.H. Chan School of Public Health; Brigham and Women’s Hospital; Broad Institute of MIT and Harvard; REgenra; UNC; Brigham and Women’s Hospital and Harvard Medical School; BWH; BWH; Framingham Heart Study Framingham MA USA; Baylor College of Medicine

**Keywords:** Metabolomics, heart failure, confounding metabolites, data-driven networks, causal network, genetic variation, lifestyle

## Abstract

In a prospective study with records of heart failure (HF) incidence, we present metabolite profiling data from individuals without HF at baseline. We uncovered the interconnectivity of metabolites using data-driven and causal networks augmented with polygenic factors. Exploring the networks, we identified metabolite broadcasters, receivers, mediators, and subnetworks corresponding to functional classes of metabolites, and provided insights into the link between metabolomic architecture and regulation in health. We incorporated the network structure into the identification of metabolites associated with HF to control the effect of confounding metabolites. We identified metabolites associated with higher or lower risk of HF incidence, the associations that were not confounded by the other metabolites, such as glycine, ureidopropionic and glycocholic acids, and LPC 18:2. We revealed the underlying relationships of the findings. For example, asparagine directly influenced glycine, and both were inversely associated with HF. These two metabolites were influenced by polygenic factors and only essential amino acids which are not synthesized in the human body and come directly from the diet. Metabolites may play a critical role in linking genetic background and lifestyle factors to HF progression. Revealing the underlying connectivity of metabolites associated with HF strengthens the findings and facilitates a mechanistic understanding of HF progression.

## Introduction

Metabolomic profiling offers a view of small molecule metabolism that reflects genetics, the environment, and their interactions. Small molecule metabolites in the blood are easily accessible and can be monitored and therefore, have great potential for gaining mechanistic insights into health and clinical translation in prevention, diagnosis, and treatment strategies. In addition, metabolite profiling may also help understand the influence of lifestyle and genetic variation on the progression to heart failure (HF), enabling precise risk prevention^1^.

In complex organisms, such as humans, metabolic pathways involve hundreds of thousands of different molecules which are interdependent. Therefore, conclusions from conventional association analysis of one or a few metabolites at a time with an outcome of interest (uni-variable or multi-variable analyses) can be confounded by other metabolites since in the classical framework of analysis, the connectivity among molecules are not considered.

The systems approach can overcome these limitations of classical approaches, where multiple molecules are analyzed simultaneously and the connectivity among them is considered in the analysis and is essential to the conclusion ^2,3^. Bayesian networks are systems approaches, data driven networks based on partial correlation. Beyond Bayesian networks are causal networks which are Bayesian networks augmented with genetic variants based on the principles of Mendelian randomization (MR). These data-driven networks provide opportunities to reconstruct biological networks, and therefore, identify mediators, confounding molecules, broadcasters, receivers and subnetworks (functional groups of molecules)^4–10^. Revealing the underlying connectivity among molecules can serve as the basis of downstream analyses, such as the identification of the effects of molecules on an outcome of interest, the effects that are not confounded by the other molecules ^4,11,12^. Moreover, the systems approach reveals the connectivity among molecules associated with outcomes which not only strengthens the findings but also provides insights into mechanistic understanding of progression to the outcome.

We previously linked a metabolic causal network of 120 metabolites to triglyceride levels^11^. It was revealed that the effects of docosahexaenoic acid and eicosapentaenoic acid (known intervention targets linked to triglyceride levels) on triglycerides are through arachidonic acid, with the largest positive effect on triglyceride levels. These findings differed from prior studies; however, a clinical validation confirmed the findings^11,13^. The validation of the novel findings using this systems approach revealed its full potential.

In a longitudinal study to investigate risk factors for cardiovascular disease, we constructed Bayesian and causal networks of metabolites at baseline. We identified the role of individual metabolites as well as groups of metabolites with a specific function (subnetworks) to provide insights into the link between metabolomic architecture and regulation in health. We incorporated the network structure into the identification of metabolites associated with HF to control the effect of confounding metabolites. In this way, we identified metabolites associated with higher or lower risk of HF incidence, the associations that were not confounded by the other metabolites. We also revealed the connectivity of metabolites associated with HF to strengthen the findings and provide insights into the link between metabolites and the progression to HF.

## Results

We analyzed 200 relative metabolite levels measured on 2,526 participants of the Framingham Heart Study (FHS) offspring cohort, free of overt HF^14^. Baseline characteristics of the study samples are provided in Supplementary Materials (Table S1). Three distinct platforms were applied to measure metabolites in the plasma samples: (1) amino acids and amines (AAA) ^15^, (2) bile acids, organic acids, nucleotides, sugars, and other intermediary metabolites (BONS) ^16^, and (3) lipids of varying classes ^17^. The list of metabolite names, including 42 AAAs, 54 BONSs, and 104 lipids, is provided in Supplementary Materials (Table S2). Genotyping was conducted for over 550,000 single nucleotide polymorphisms (SNPs) ^18^. The participants were followed up and the first HF incident was recorded.

Using systems approaches, we here analyzed these data and presented two key aspects of the analyses: first, characterizing interconnectivity of metabolites and revealing them as networks, and second, incorporating the network structure to identify the effect of metabolites on HF incidence. For the former, we focused on metabolites measured in each platform and identified three data-driven networks of AAA, BONS, and lipids, where a link represents a partial correlation, a correlation that is not attributable to the other metabolites in the network. To identify causal networks, we augmented the networks with polygenic factors, generated by extracting information from genetic variations. Some polygenic factors satisfied MR assumptions to facilitate direction identification in the AAA network. However, there was not such polygenic factors for BONS/lipid networks. We extracted network properties and revealed the role of each metabolite individually and subnetworks.

**Figure 1:**
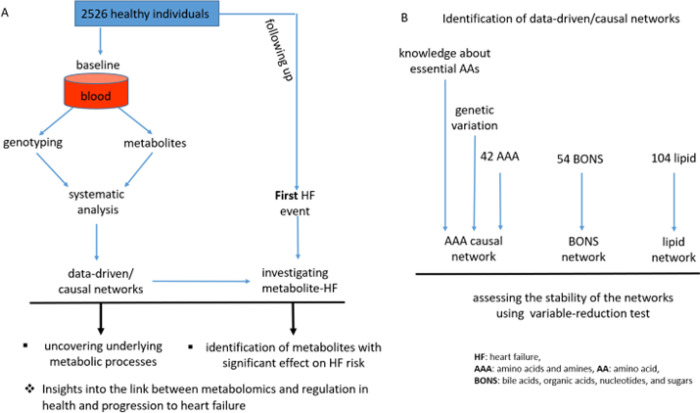
Study design and analysis workflow. **A. Data acquisition process and overall study design.** The data were obtained from participants in the offspring cohort of the Framingham Heart Study (FHS) who were free of overt HF at baseline and followed for the first of HF incidence, death, or last follow up contact free of HF. **B. The identification of data-driven/causal networks.** Some polygenic factors (generated by extracting information from genetic variations) satisfied the MR assumptions and facilitated the identification of the AAA causal network, along with the knowledge that the essential AAs are influenced only by exogenous factors (lifestyle (e.g., diet) and genetic variation). Abbreviations, HF: heart failure, AAA: amino acids and amines, AA: amino acid, BONS: bile acids, organic acids, nucleotides, and sugars.

In the second aspect of the analysis, we linked the networks to HF incidence. We used a model that was controlled for the effect of confounding metabolites in addition to sex, age, and BMI. For each metabolite, we provided 4 properties: effect size, significant level (*p*-value), hazard ratio, and connectivity with the other metabolites. Fig. 1 illustrates the study design and analysis workflow. The association of HF incidence with each single metabolite without considering the possible confounding by other metabolites are provided in (Tables S3-S8).

### First aspect: Metabolomics and insights into regulation in health

#### Uncovering AAA connectivity and distinguishing broadcasters from receivers.

**Figure 2:**
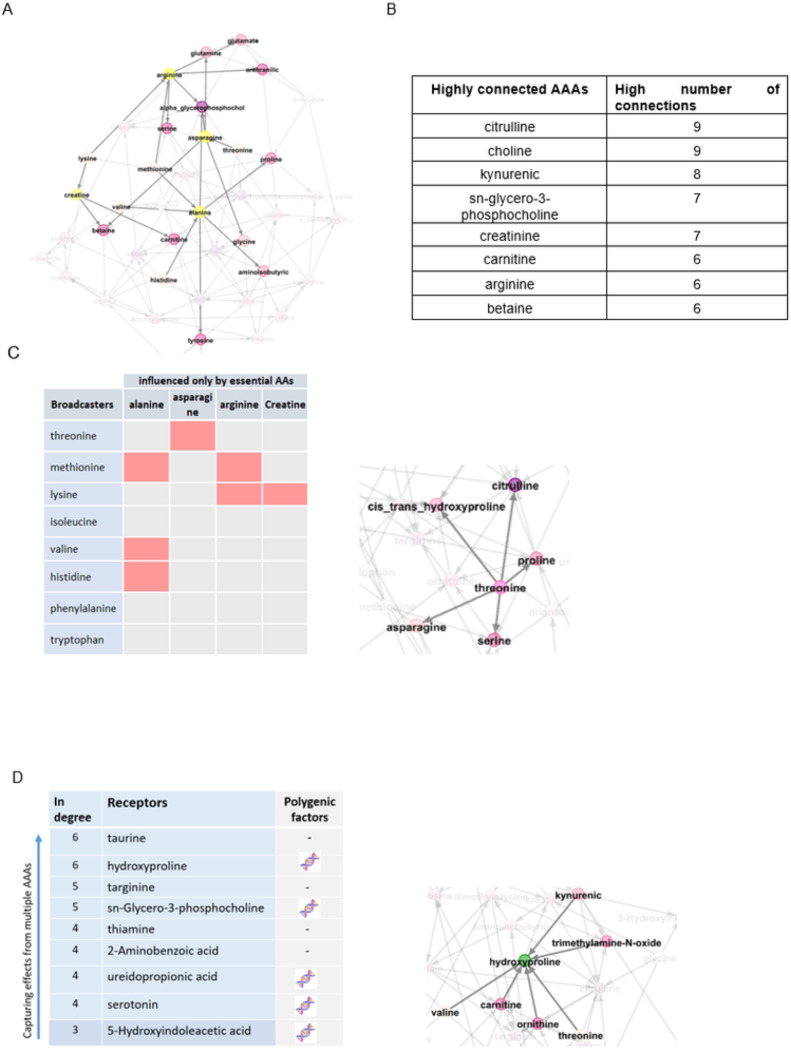
AAA causal network. **A.** The AAA network with a focus on the four metabolites influenced only by essential AAs. These four metabolites are depicted in yellow, and their neighbors are depicted in light to dark pink based on the number of *in degree. In degree* represents the number of metabolites that affect the indicated metabolite. **B.** AAAs highly connected with other AAAs (>= 6, regardless of directionality). For example, citrulline is connected with 9 other AAAs either influences or is influenced by them. **C.** The AAA broadcasters, metabolites directly affecting multiple other metabolites. *Out degree* represents the number of metabolites affected by the indicated metabolite. The four metabolites on top (arginine, asparagine, alanine, and creatine) were influenced only by essential AAs and not by the other metabolites. The connectivity of these four metabolites with essential AAs are depicted with pink boxes, e.g., lysine affects arginine and creatine. Threonine as a broadcaster is depicted with the neighboring connectivity. **D.** The AAA receivers, metabolites highly influenced by the other AAAs and with no or very low impact on others. The DNA double helix depicts metabolites that were directly influenced by polygenic factors in a conditional independence network. Hydroxyproline, as an example of a receiver, is depicted along with direct network neighbors.

In total, 8 out of 42 AAAs were directly influenced by polygenic factors that satisfied Mendelian randomization assumptions in the AAA causal network. The list of these metabolites is provided in the supplementary, (Table S9). In constructing the AAA causal network, we assumed that the exogenous variables (lifestyle/genetic variation) are the only sources of variation in essential AAs (threonine, lysine, isoleucine, valine, methionine, histidine, phenylalanine, tryptophan, and leucine). The AAA network is depicted in Fig. 2A with a focus on four metabolites (arginine, asparagine, alanine, and creatine) influenced only by essential AAs.

The connectivity/link between each two AAAs were highly significant (*p*-value < 1´10^−15^). In addition, some of AAAs had a high number of connectivity (>= 6), i.e., directly connected to several metabolites, (Fig. 2B). Some of the AAAs were “broadcasters”^4^, i.e., metabolites directly affecting multiple metabolites with effects propagated downstream to other metabolites, (Fig. 2C); threonine, as an example of a broadcaster, affects 5 other metabolites directly (*out degree* = 5) and through each of them, affects multiple other metabolites indirectly. Unlike broadcasters, some metabolites were “receivers”, i.e., metabolites with no or very low impact on other metabolites but receiving impact from multiple other metabolites, (Fig. 2D); hydroxyproline, as an example of a receiver, is influenced by 6 other metabolites directly (*in degree* = 6).

#### Uncovering BONS connectivity and distinguishing broadcasters from receivers.

**Figure 3:**
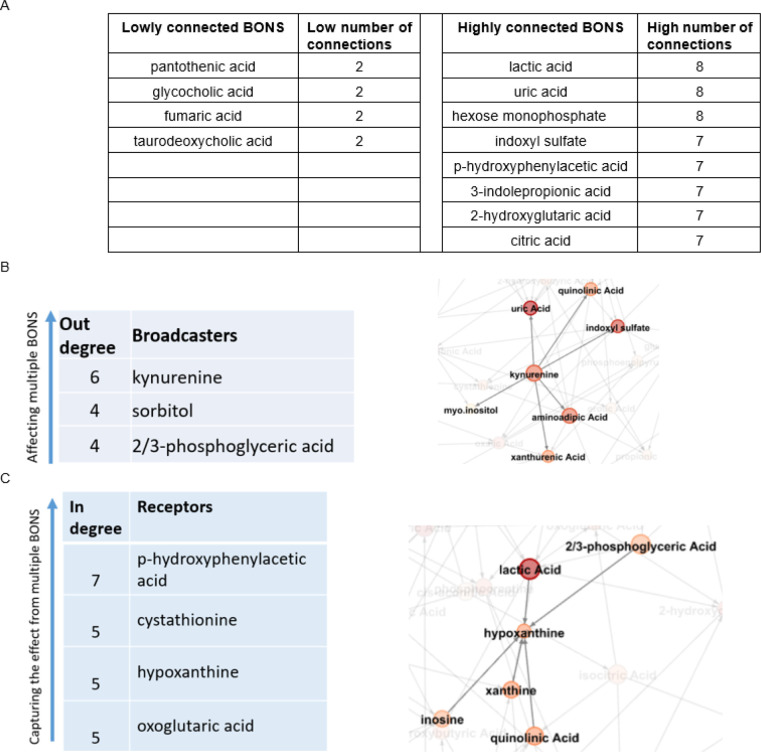
BONS data-driven network. **A.** Metaboliteswith highest and lowest connectivity. For example, lactate is highly connected to other metabolites (connectivity = 8), while pantothenic acid is connected to only 2 other metabolites (connectivity = 2). **B.** The broadcasters. The effects of these metabolites propagate to multiple other metabolites. **C.** The receivers. Metabolites with no or very low impact on others but highly influenced by the other BONS metabolites.

In the data-driven network of 54 BONS, we observed some metabolites with high connectivity (>=7) and some with very low connectivity (<= 2) (Fig. 3A). The hexose monophosphate among the high connected metabolites belongs to the carbohydrate super pathway and the glycolysis sub-pathway and is composed of fructose-1-phosphate, fructose-6-phosphate, glucose-1-phosphate, and glucose-6-phosphate. More information about this group of metabolites is provided in (Supplementary Materials).

Some of BONS metabolites had the property of broadcasters, affecting multiple other metabolites directly, as illustrated by kynurenine as an example (Fig. 3B). In contrast, there were some receivers, BONS metabolites with no or very low impact on other metabolites but highly influenced by others, as illustrated by hypoxanthine as an example (Fig. 3C).

#### Uncovering lipid connectivity and distinguishing broadcasters from receivers as well as subnetworks.

**Figure 4:**
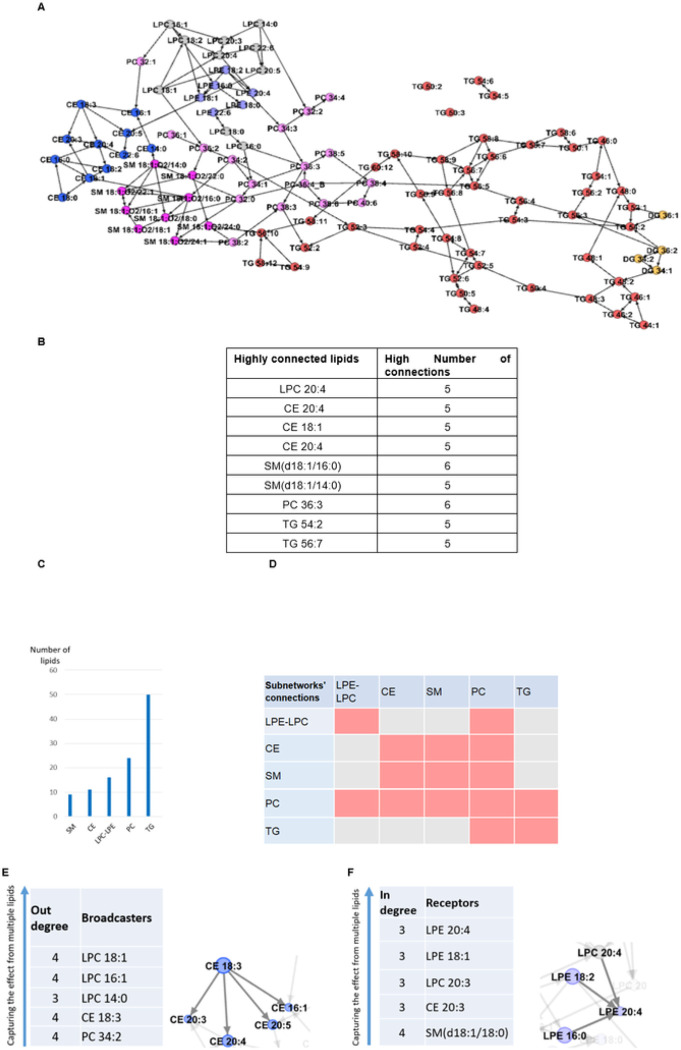
Lipid data-driven network. **A.** The lipid network, key mediators, and subnetworks. Five subnetworks, lysophosphatidyl ethanolamine and lysophosphatidyl choline (LPE-LPC), cholesteryl ester (CE), sphingomyelin (SM), phosphatidylcholine (PC), as well as triacylglycerol and diacylglycerol (TG) were identified, depicted in different colors. There were only 4 diacylglycerol lipids, depicted as yellow nodes. They were connected only to TGs but not influenced by them. The PC lipids were mediators between TG lipids and the others. The CE and TG had almost no impact on other lipids. While the CE lipids were not influenced significantly by others either, TG lipids were (*p*-value < 1´10^−4^). **B.** Lipids with high connectivity (>= 5), e.g., LPC 20:4 was connected to 5 other lipids. **C.** The number of lipids in each subnetwork. **D.** The connectivity between lipid subnetworks, e.g., the PC subnetwork was connected to all other subnetworks. But SM lipids were connected to CE and PC. **E.** Lipid broadcasters inside subnetworks. The connectivity of CE 18:3 as an example of a broadcaster is depicted. **F.** Lipid receivers inside subnetworks. The connectivity of LPE 20:4 as an example of a receiver is depicted. Note that there are no broadcasters and no receivers in the TG subnetwork.

In the data-driven network of 104 lipids, we observed five subnetworks corresponding to structural classes of metabolites, lysophosphatidylethanolamine and lysophosphatidylcholine (LPE-LPC), cholesteryl ester (CE), sphingomyelin (SM), and phosphatidylcholine (PC), as well as triacylglycerol and diacylglycerol lipids (TG) (Fig. 4A). We extracted lipids with high connectivity (>=5) (Fig. 4B). The number of lipids in each subnetwork is provided in Fig. 4C.

The connections within LPE-LPC, CE, and SM subnetworks were highly significant (*p*-value < 5´10^−35^). We observed that PC lipids were mostly mediators to spread the effect of the LPE-LPC lipids to other subnetworks (Fig. 4A and 4D). There was no individual lipid with broadcaster or receiver properties across the network; however, with the exception of TG, some lipids within each subnetwork demonstrated such properties (Fig. 4E–F).

### Second aspect: Link between metabolites and the progression to HF

**Figure 5:**
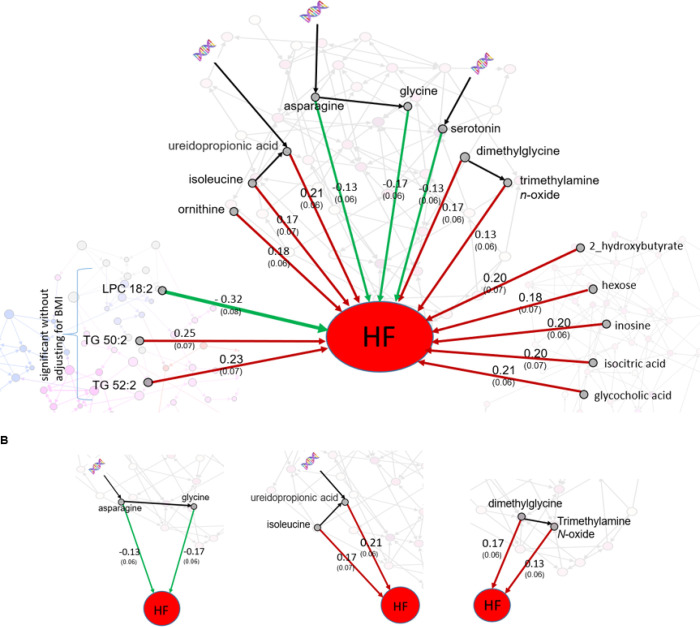
**A. Metabolites associated with HF risk after controlling for confounding metabolites.** The green/red arrows represent the effect of metabolites with decrease/increase in HF risk. The double helix represents metabolites directly influenced by polygenic factors in the conditional independence networks. The numbers on arrows represent effect size (standard error). The associations of AAA and BONS metabolites with HF were after adjusting for confounding metabolites, age, sex and BMI. The three lipids, however, were associated with HF risk after adjusting for confounding metabolites, age, and sex but not BMI. **B. Zooming in the connections between metabolites associated with HF.** Asparagine directly affects glycine, and both reduce HF risk. Isoleucine directly affects ureidopropionic acid, and both elevate HF risk. We observed the same property for dimethylglycine and trimethylamine N-oxide.

We identified metabolites associated with future HF risk after controlling for the confounding metabolites and provided four properties for each metabolite including hazard ratio, effect size, the significance level (*p*-value), and connectivity (Table 1, Fig. 5).

Out of 8 AAAs associated with HF incidence, three were inversely associated, including glycine, asparagine, and serotonin, and all three were associated with polygenic factors. Asparagine was also directly affected by the essential amino acid threonine, and in turn, asparagine directly affects glycine.

Isoleucine and ureidopropionic acid are among the five AAAs associated with increased risk of HF incidence. In the AAA causal network, we observed that isoleucine affected ureidopropionic acid directly, and in turn both contributed to an increase in HF risk (Fig. 5B). Note that the effect of ureidopropionic acid is not attributed to isoleucine. While assessing the effect of ureidopropionic acid on HF incidence, isoleucine is considered a confounder and was included in the Cox model. However, the effect of ureidopropionic acid on HF risk remained significant even after adjusting for isoleucine. The same property was observed for dimethylglycine and trimethylamine N-oxide (Fig. 5B).

Five BONSs were associated with HF incidence: glycocholic acid, inosine, 2-hydroxybutyrate, isocitric acid, and hexoses (hexose metabolites, fructose-glucose-galactose). All of these were directly associated with HF incidence (Table 1). We revealed metabolites directly connected to isocitric acid which may facilitate understanding the unknown mechanisms of isocitric acid association with HF (Supplementary, Fig. S1). The relatively higher *out degree* of both hexose and isocitric acid (*out degree* = 5) suggests that there are multiple other BONSs that may increase HF risk, but their p-values did not pass the statistical significance threshold. The *out*/*in degree* of glycocholic acid was not identified because the direction of links directly connected to it were not identified. The snapshot of the network with a focus on glycocholic acid is provided in Supplementary (Fig. S2).

After adjusting for confounding lipids, age, sex, and BMI, no lipid was identified associated with HF incidence. However, adjusting for confounding lipids, age, sex, three lipids were associated with HF risk, among them LPC 18:2 had a negative effect on HF (Table 1).

## Discussion

In this study, data-driven/causal networks of metabolites were identified based on conditional independence structure and MR principles. Revealing the underlying connectivity among metabolites served as the basis of finding metabolites with significant effects on HF, the effects that were not confounded by the other metabolites.

The link between each two metabolites in the network, was not attributed to the other metabolites. The connectivity among metabolites provided information on the biological dependencies of metabolites which facilitates gaining mechanistic insights into the link between metabolites and regulation in health. For example, four metabolites (arginine, asparagine, alanine, and creatine) were influenced only by essential AAs and not by the other metabolites. These four metabolites and essential AAs were broadcasters, i.e., influenced all other metabolites in the study directly or indirectly. Therefore, we conclude that any change in these metabolites (e.g., drug or lifestyle) would be expected to change the levels of the other metabolites. Some metabolites were identified as receivers (e.g., hydroxyproline and taurine), with no or very low impact on other metabolites. However, they were influenced by multiple other metabolites. Therefore, we conclude the levels of these metabolites can predict the levels of the other metabolites.

After finding metabolite-HF associations, we revealed the connectivity among these metabolites associated with HF. This not only strengthens the findings but also provides insights into mechanisms of progression to HF. There were three AAAs (glycine, asparagine, and serotonin) associated with a decrease in HF risk where all three were also associated with polygenic factors (serotonin, and asparagine directly and glycine after one step through asparagine). Asparagine was also directly affected by the essential amino acid threonine not synthesized in the human body so comes directly from diet. In the metabolomic network, we observed that asparagine affects glycine directly, a link that is not attributed to other metabolites (Fig. 5B). These metabolites may play a critical role in linking genetic background and diet to lowering HF risk. The causal association of circulating levels of glycine with lower incidence of coronary heart disease has previously been found using genetic variations^19^. Our findings here strengthen evidence for a protective effect of glycine on heart disease and also provide insights in the related mechanisms.

Asparagine affects glutamine directly, and the effect was not attributed to any other metabolites in the study. Multiple studies have demonstrated that glutamine supplementation protects against cardiometabolic disease, ischemia-reperfusion injury, cardiac injury by inimical stimuli, and may be beneficial in patients with heart failure^20–23^. Our findings, including the direct effect of asparagine on glutamine and the effect of asparagine on lowering HF risk, which was not confounded by the other metabolites, may facilitate understanding the underlying mechanisms.

We observed that trimethylamine *N*-oxide is directly associated with HF incidence. This metabolite was a carnitine metabolite associated with gut microbiota and carnitine processing associated with HF risk and atherosclerosis^24–26^. Elevated trimethylamine *N*-oxide was found to preserve mitochondrial energy metabolism and cardiac functionality in a rat model of right ventricular HF^27^. Here, we observed that trimethylamine *N*-oxide was directly affected by dimethylglycine, which was a confounder of the effect of the effect trimethylamine *N*-oxide on HF. However, after adjusting for the effect of dimethylglycine, the effect trimethylamine *N*-oxide on HF remained significant. The findings here may illuminate underlying mechanisms of their effects on HF risk growth.

All BONSs were directly associated with HF incidence. Excess bile acids have been reported to decrease fatty acid oxidation in cardiomyocytes leading to heart dysfunction^28^. One of BONSs associated with HF risk was 2-hydroxybutyrate. Hydroxybutyrate was important for energy production in humans. Enhanced cardiac blood flow and lowered myocardial glucose uptake were observed by an infusion of hydroxybutyrate in healthy individuals^29^. Inosine was another BONS associated with HF risk which was previously identified as a biomarker for cardiac ischemia^30^. Isocitric acid was previously found associated with reduced ejection fraction^31^. The exact mechanism underlying this observation was not known. To facilitate the mechanistic understanding, we depicted the underlying connectivity of isocitric acid with other BONSs (Supplementary, Fig. 1).

Essential AAs are not synthesized in the human body and come directly from diet or lifestyle. The lifestyle source of variation in metabolites and environmental pollutants can vary widely between different populations. The participants in this study were European Americans, which may have influenced the metabolite levels. We analyzed metabolites in each platform separately. Therefore, metabolite-HF associations in each platform have not been adjusted for the effect of confounding metabolites from the other platforms if there were any. The covariates considered here were sex, age, and BMI, the covariates with highest impacts on HF. However, in some studies other covariates are considered too.

In this study, we provided insights into metabolomics, such as finding metabolites functioning as receivers and broadcasters across AAA and BONS networks. Lipids were grouped in 5 subnetworks and each subnetwork had its own receivers and broadcasters. We identified metabolites with effects on HF risk reduction and elevation. Some AAA metabolites linked genetic and diet to HF risk reduction. However, BONS metabolites were only associated with HF risk elevation. These effects were not attributed to other metabolites since the effect of confounding metabolites were adjusted. We provided four properties for each metabolite associated with HF including hazard ratio, effect size, the significance level (*p*-value), and connectivity. Revealing connectivity among metabolites strengthens the findings and facilitates mechanistic understanding of HF progression. The HF risk associated metabolites identified in this study provide a prioritized list of candidate metabolites from a large set of metabolites, now suitable for designing additional, more focused-experimental studies to uncover pathways reflecting the progression to HF.

## Methods

### Study cohort:

In 1948, a sample of residents from Framingham, Massachusetts, U.S. participated in a longitudinal study to investigate risk factors for cardiovascular disease. The study was expanded in 1971, by the Framingham Offspring cohort, a sample of children (including their spouses) of the Framingham Original cohort participants. The participants of the FHS, Offspring Cohort, have been followed in examination cycles of 4 to 6-year intervals for the onset of cardiovascular disease. All participants from the Offspring Cohort had blood drawn after overnight fasting during the fifth examination cycle (undertaken in 1991–1995), which we consider here baseline. Blood was stored at −80 C and used for metabolomic profiling between 2009 and 2011 using liquid chromatography with mass spectrometry techniques^15^. In addition, common single nucleotide polymorphisms (SNPs) were genotyped using approximately 550,000 SNPs (Affymetrix 500K mapping array plus Affymetrix 50K supplemental array)^32^ available in the database of genotypes and phenotypes (dbGap Study Accession: phs000007.v32.p13)^18^. The focus of this study was on records from 2,526 individuals (mean age 55 ± 10 years, 48% men) free of HF at baseline (the cycle that blood was drawn and isolated plasma was used for untargeted metabolomic profiling)^33^.

HF was defined according to the established FHS criteria outlined elsewhere^34^. All participants were under continuous surveillance for incident HF by the review of hospitalization charts and by patient history and physical examinations during the FHS clinic visits. For all HF events, the final adjudication was undertaken by an expert study committee comprising at least three FHS physicians, of whom at least two were cardiologists. We measured the time to HF event as the time that the blood of each person was taken until the time of HF diagnosis. For the participants who did not develop HF, censoring time was at death or last follow up contact free of HF. We considered incident HF events developed through Dec 31, 2016, which was between 20–25 years after the blood samples were taken. In total, 260 HF events were recorded as the first HF event, which occurred, on average, more than 10 years after baseline, when metabolites were obtained.

### Metabolic networks:

The metabolites were log-transformed to be normally distributed and any remaining outliers with skewed distributions were winsorized by shrinking values beyond a cutoff of 3 standard deviations ± the mean. Then, metabolites were adjusted for batch, age, sex, and BMI effects. The networks were then constructed on the residuals. We estimated a network model of conditional dependencies, where edges represent partial correlation between two metabolites, a correlation that could not be explained by any subset of other metabolites. To estimate the networks, we applied an order-independent implementation of the conditional independence structure, i.e., directed acyclic graph (DAG), learning PC-algorithm^35^. In the next step, we generated and augmented the networks with polygenic factors using the G-DAG algorithms to identify causal networks established in the MR principles. These steps have been reviewed previously in multiple publications^4,5,11,36–38^ and a brief review is provided in Supplementary Materials. Polygenic factors are extracted information from genetic variants (here, by using principal component analysis) and used as instrumental variables, introduced by Yazdani et al. (2016)^36^. This approach was followed by others, such as Burgess et al. (2017)^39^.

Each polygenic factor explains a large amount of genetic variation. Polygenic factors prevent spurious estimates and inflated Type 1 error when using too many genetic variants in the analysis or highly sensitive estimates due to ignoring most data and using a few genetic variants. In addition, the results of the MR approaches using polygenic factors are more robust to seemingly arbitrary choices in the variable selection step (Yazdani et al., 2022^38^).

The threshold for constructing robust networks of metabolites was a = 1´10^−4^ obtained by minimizing structural Hamming distance^40,41^. Out of the 257 generated polygenic factors, 37 factors satisfied the MR assumptions for AAA metabolites (i.e., valid instrumental variables) and therefore, used for the identification of the AAA causal network. We also extended the G-DAG algorithm to include the knowledge that the sources of variation in essential AAs are exogenous, which means any identified edge from an essential AA to another metabolite were directed from the former to the latter. For the BONS and lipid metabolites, no polygenic factors were identified to satisfy the MR assumptions and there was no established prior knowledge. However, multiple directions were identified based on v-structure property^42^, (for details see Supplementary Materials). After identification of the networks, we applied the variable-reduction test to each network to assess the stability of the directions^38^ (Supplementary). If some directions were not stable, we did not remove the corresponding metabolites from the study, but instead, we considered bi-directed edges between them as unidentified directions.

### Identification of confounding metabolites using the network:

**Figure 6:**
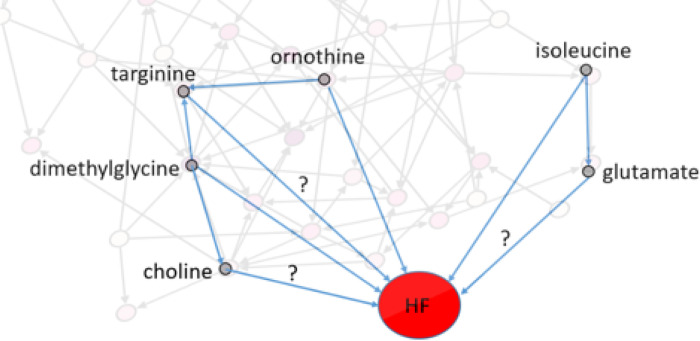
Confounding metabolites. Some of the metabolite-HF associations and the confounding metabolites are depicted here. Isoleucine and dimethylglycine are confounders of glutamate and choline associations with HF respectively. dimethylglycine and ornithine both are confounders of targinine association with HF. In Fig. 2A, the arrow from isoleucine on glutamate was not highlighted since it was not the focused there.

Confounding metabolites are defined here as those that affect both the metabolite of interest and HF^43,44^. The effect of metabolites on each other was considered from the network (*p*-value < 1´10^−4^) and the effect of metabolites on HF was considered from uni-variable analysis (*p*-value < 5´10^−2^). For example, the effect of isoleucine on HF risk is not confounded because it is an essential AA, and no metabolite is affecting it. Isoleucine is associated with HF (*p*-value < 5´10^−2^) and therefore, is a confounder for association of HF risk and any metabolite of interest that is connected to isoleucine, such as glutamate and ureidopropionic acid (Fig.6). Another example is the effect of targinine on HF. The two metabolites ornithine and dimethylglycine affect targinine. Both ornithine and dimethylglycine are associated with HF. Therefore, these two metabolites are the confounding metabolites for the effect of targinine on HF risk.

### Cox regression models:

To investigate the effect of a metabolite on incident HF, we applied the Cox proportional hazards model after confirming that the assumption of proportionality of hazards was met. The Cox models were applied to normalized metabolites. We measured the time to HF incidence from the time that the blood of each person was taken (the time that metabolites were measured/baseline) until the time of HF diagnosis.

We integrated the structural equation modeling with Cox models. The assumption of application of structural equation modeling is the confounders are included in the structural equation; in other word, the equations are based on the underlying structure, which here was revealed by the networks. Therefore, to satisfy the assumption, we adjusted the Cox models not only for the set of covariates, but also for the confounding metabolites:

$$ln\;(H(t;(X,Z)))=ln\left(h_{0}(t)\right)+\theta X+\beta Z,$$

where H(t) stands for cumulative hazard, is a time-dependent baseline hazard term, X and Z are the vectors of covariates and metabolites respectively, and and are matrixes of coefficients of covariates and metabolites respectively.

The confounding metabolites are considered those that are associated with HF at level (*p*-value < 0.05) in a uni-variable analysis. We identified metabolite-HF associations after adjusting for the effect of confounding metabolites as well as covariates. Then, metabolite-HF associations are considered significant at p-value < 9 ´10^−3^, equivalent with false discover rate (FDR)-adjusted *p*-value of 0.1 (Benjamini-Hochberg method).

## Figures and Tables

**Table 1.** Metabolites with significant effect on HF risk after controlling for confounding metabolites. A higher *out degree* represents a higher impact of the indicated metabolite on the other metabolites. A higher *in degree* represents a higher impact of other metabolites on the indicated metabolite. Glycine and isoleucine had the highest impact on other AAAs, *out degree* = 4, among metabolites associated with HF risk. The out/in *degree* of glycocholic acid was not identified because the direction of links directly connected to it were not identified. Three lipids showed association with HF risk after adjusting for sex and age; adjusting for BMI in addition to age and sex, no lipid was associated with HF risk.

AAA	Hazard |Ratio|	Effect size	S.E.	*p*-value	Connectivity	
					*Out degree*	*In degree*
ureidopropionic acid	1.24	0.21	0.06	1×10^−3^	0	2
ornithine	1.20	0.18	0.06	5×10^−3^	2	4
glycine	0.84	−0.17	0.06	9×10^−3^	4	1
dimethylglycine	1.18	0.17	0.06	1×10^−2^	2	4
isoleucine	1.17	0.17	0.07	2×10^−2^	4	0
asparagine	0.87	−0.13	0.06	4×10^−2^	3	1
trimethylamine *N*-oxide	1.14	0.13	0.06	4×10^−2^	1	3
serotonin	0.88	−0.13	0.06	4×10^−2^	0	2
**BONS**						
glycocholic acid	1.23	0.21	0.06	1×10^−3^	0 or 2	0 or 2
inosine	1.22	0.20	0.06	3×10^−3^	2	2
2-hydroxybutyrate	1.22	0.20	0.07	5×10^−3^	2	3
isocitric acid	1.22	0.20	0.07	6×10^−3^	4	1
hexose	1.20	0.18	0.07	9×10^−3^	4	2
**Lipid**						
LPC 18:2	0.71	−0.32	0.08	9×10^−5^	2	2
TG 50:2	1.28	0.25	0.07	4×10^−4^	0	0
TG 52:2	1.26	0.23	0.07	1×10^−3^	0	2
